# The economics of the energy transition

**DOI:** 10.1007/s13209-021-00238-4

**Published:** 2021-06-02

**Authors:** Natalia Fabra, Xavier Labandeira

**Affiliations:** 1 Universidad Carlos III de Madrid, Madrid, Spain; 2 Universidad de Vigo, Vigo, Spain

The Energy Transition is underway. An increasing number of countries—with Europe, and now the USA, leading the way—have committed to drastically reducing their emissions during the coming decades. The European Green Deal, that was announced in Madrid in December 2019 just before the COVID-19 hit our economies, was the first of a series of commitments to reach carbon neutrality no later than 2050. The European Recovery and Resilience Plan, with its green and digital conditionality, has backed this ambition by providing funds which will accelerate the achievement of this goal. All sectors of the economy—with no exception—will be impacted by this challenge: power, transportation, construction or agriculture, to name just a few, will go through profound structural changes which will bring in opportunities, but also risks and challenges. Likewise, the Energy Transition will have deep socio-economic implications, which will broadly depend on the set of regulatory and tax policies that will be put in place.

Economics has to play a key role in the Energy Transition. It has to guide policy-making in this area by identifying the obstacles that may hamper or delay the Energy Transition. It also has to provide guidance on how best to design policies that contribute to reducing the costs of the Energy Transition, while mitigating any adverse distributional implications. Last, but not least, it has to make use of its quantitative toolkit to measure the impacts that the Energy Transition will have on our economies and societies.

The overarching objective of decarbonizing all sectors of the economy calls researchers in this area to multiply their efforts over the next few years. This was indeed the guiding motivation of the Journal of the Spanish Economic Association-SERIEs for launching a Special Issue on the Economics of the Energy Transition. The topics that were proposed in the call for papers reflected the broad diversity of research questions and challenges ahead: from the design and effects of climate mitigation policies to the role of companies in the Energy Transition, from the thorough analysis of the distributional impacts of the Energy Transition to its impacts in terms of economic growth or job creation. This is coupled with the richness of methodologies that are used to explore these issues, including theoretical, empirical and simulation approaches.

Although some of the aforementioned suggested topics did not materialize in actual submissions, five papers on related matters were eventually selected. We are very grateful to the colleagues who responded to our call for providing pieces of high-quality research. We also have a big debt toward the reviewers who helped us in taking the final decisions, in some cases upon short notice and close deadlines. They undoubtedly contributed to making this Special Issue better. Last but not least, this issue would not exist without the continuous support and interest of Virginia Sánchez-Marcos, the editor-in-chief of the journal.

This Special Issue starts with the paper by Luis Puch, Gustavo Marrero, Josué Barrera and Antonia Díaz, which is very much at the heart of the topics that motivated our call: What has been the path toward the green transition in Europe so far? In particular, how has this transition differed across countries and why? To answer these questions, the authors use data from sixteen western European countries for a forty-year period (1980–2019). As their main conclusion, they report a strong relationship between economic activity and carbon emissions in economies where economic booms depend on energy-intensive sectors. Puch et al. further strengthen the key role that renewable energy technologies play in mitigating the adverse effects from energy intensity rebounds. This is a very timely paper as it can provide light on the recovery challenge.

The paper by Darío Serrano-Puente also provides empirical evidence on the transition to decarbonization in Europe, with a specific focus on the Spanish situation in EU terms. In particular, the article uses a decomposition method to assign the responsibility of primary energy requirements (related to carbon dioxide emissions) to end-use sectors within the Spanish economy. Darío reports that energy efficiency has been improving in Spain since the great recession, even though some of the gains have been offset by an inefficient use of the installed energy equipment. Although Spain is improving in this area at a greater pace than the EU average, Darío stresses that “there is much to be done” due to the large increase in emissions that took place before 2007.

Energy efficiency is indeed crucial for a successful transition to a decarbonized society, and this is also the departing point of the paper by Carmen Arguedas and Sandra Rosseau. They approach it by exploring the relationship between public policies aimed at improving energy efficiency and firms’ strategies (e.g., regarding product design). Their analysis stresses the pivotal role that consumers can play in promoting environmental improvements at the firm level. In particular, Carmen and Sandra emphasize the importance of the interactions between direct and indirect actions by the regulator and the environmental awareness of consumers under different market structures.

Ángela García-Alaminos and Santiago Rubio provide another theoretical inquiry on the optimal policy mix to deal with a monopoly that produces a good with a polluting input and a clean technology. The authors show that the efficient solution can be achieved by combining a tax on emissions, which is lower than the value of environmental damages, plus a subsidy on the clean output. The second-best tax, which in certain cases should be set at the Pigouvian level, and the second best subsidy, are found by solving a two-stage policy game between the regulator and the monopoly firm.

Finally, Georg Zoettl deals with an increasingly popular policy instrument to promote climate mitigation: cap and trade for CO2 emissions. He argues that one of the reasons for such popularity is the possibility to allocate free carbon permits to emitters—so-called, grandfathering—in order to mitigate their cost burden, particularly in sectors exposed to international competition. Yet, unlike most existing analyses, Georg considers free permit allocations that are not lump-sum but contingent on firms’ actions (e.g., regarding entry and exit). Although some papers have analyzed the output distortions brought about by grandfathering, Georg focuses on its effects on the incentives to invest in different electricity generation technologies. His papers thus sheds light on how to better design cap and trade systems in this context.

